# Multicenter, Phase III, Randomized, Double-Blind, Placebo-Controlled Trial of Pravastatin Added to First-Line Standard Chemotherapy in Small-Cell Lung Cancer (LUNGSTAR)

**DOI:** 10.1200/JCO.2016.69.7391

**Published:** 2017-02-27

**Authors:** Michael J. Seckl, Christian H. Ottensmeier, Michael Cullen, Peter Schmid, Yenting Ngai, Dakshinamoorthy Muthukumar, Joyce Thompson, Susan Harden, Gary Middleton, Kate M. Fife, Barbara Crosse, Paul Taylor, Stephen Nash, Allan Hackshaw

**Affiliations:** Michael J. Seckl, Imperial College London; Yenting Ngai, Stephen Nash, and Allan Hackshaw, Cancer Research UK and University College London Cancer Trials Centre; Christian H. Ottensmeier, University of Southampton and Southampton University Hospitals, Southampton; Michael Cullen, Queen Elizabeth Hospital Birmingham; Joyce Thompson, Heart of England Birmingham; Gary Middleton, University of Birmingham, Birmingham; Peter Schmid, Brighton and Sussex Medical School, Brighton; Dakshinamoorthy Muthukumar, Colchester Hospital, Colchester; Susan Harden, Cambridge University Hospital, Cambridge; Kate M. Fife, Peterborough City Hospital, Peterborough; Barbara Crosse, Calderdale and Huddersfield NHS Foundation Trust, Huddersfield; and Paul Taylor, University Hospital South Manchester, Manchester, United Kingdom.

## Abstract

**Purpose:**

Treating small-cell lung cancer (SCLC) remains a therapeutic challenge. Experimental studies show that statins exert additive effects with agents, such as cisplatin, to impair tumor growth, and observational studies suggest that statins combined with anticancer therapies delay relapse and prolong life in several cancer types. To our knowledge, we report the first large, randomized, placebo-controlled, double-blind trial of a statin with standard-of-care for patients with cancer, specifically SCLC.

**Patients and Methods:**

Patients with confirmed SCLC (limited or extensive disease) and performance status 0 to 3 were randomly assigned to receive daily pravastatin 40 mg or placebo, combined with up to six cycles of etoposide plus cisplatin or carboplatin every 3 weeks, until disease progression or intolerable toxicity. Primary end point was overall survival (OS), and secondary end points were progression-free survival (PFS), response rate, and toxicity.

**Results:**

Eight hundred forty-six patients from 91 United Kingdom hospitals were recruited. The median age of recruited patients was 64 years of age, 43% had limited disease, and 57% had extensive disease. There were 758 deaths and 787 PFS events. No benefit was found for pravastatin, either in all patients or in several subgroups. For pravastatin versus placebo, the 2-year OS rate was 13.2% (95% CI, 10.0 to 16.7) versus 14.1% (95% CI, 10.9 to 17.7), respectively, with a hazard ratio of 1.01 (95% CI, 0.88 to 1.16; *P* = .90. The median OS was 10.7 months *v* 10.6 months, respectively. The median PFS was 7.7 months *v* 7.3 months, respectively. The median OS (pravastatin *v* placebo) was 14.6 months in both groups for limited disease and 9.1 months versus 8.8 months, respectively, for extensive disease. Adverse events were similar between groups.

**Conclusion:**

Pravastatin 40 mg combined with standard SCLC therapy, although safe, does not benefit patients. Our conclusions are the same as those found in all four much smaller, randomized, placebo-controlled trials specifically designed to evaluate statin therapy in patients with cancer.

## INTRODUCTION

Small-cell lung cancer (SCLC) accounts for 15% to 20% of all new cases of lung cancer worldwide, with a low median survival of 12 to 14 months for patients with limited stage disease and 8 to 12 months for those with extensive stage disease. Although few therapeutic advances have been made over the past 40 years, there has been much progress in the understanding of the biologic processes, including the importance of, for example, TP53 and RB1 gene mutations, and the potential for targeted therapies—for example, poly (ADP-ribose) polymerase inhibitors and immunotherapeutics—of which several trials are ongoing.^1^

Statins are an inexpensive and established therapy for cardiovascular disease prevention and treatment. Despite initial concerns that long-term use might increase the risk of developing cancer, large-scale meta-analyses of randomized trials have shown no excess cancer incidence or mortality^2^; however, evidence from experimental and preclinical studies has indicated that statins can inhibit tumor growth and induce apoptosis in several tumor types, including pancreatic carcinoma,^3^ mesothelioma,^4^ breast cancer,^5^ and SCLC cells.^6^ Mechanistically, mitogen-activated protein kinase and extracellular signal-regulated kinase upregulates antiapoptotic molecules in SCLC cells,^7,8^ and simvastatin can disrupt this process through impaired Ras superfamily signaling. This is achieved because statins block 3-hydroxy-3-methyl-glutaryl-coenzyme A (HMG-CoA) reductase, thereby reducing cholesterol biosynthesis and impairing geranylation and farnesylation of Ras superfamily members.^6^ Statins could therefore act in an additive or synergistic fashion when combined with chemotherapy agents, such as paclitaxel,^9^ cisplatin,^5,10^ and doxorubicin.^5^ Experiments in H-69 human SCLC xenografts in nude mice showed impressive single-agent activity of orally administered statin.^6^ Our early studies implied that statins, such as simvastatin, would enhance the effects of single-agent or combination chemotherapy, including cisplatin and etoposide, triggering apoptosis and/or reverse chemoresistance.^6^ These effects were not confined to one platinum type because atorvastatin has subsequently been shown to enhance efficacy of carboplatin in non-SCLC cells in vitro and in vivo.^11^ Furthermore, before our trial, an unblinded randomized trial of pravastatin in patients with liver cancer reported a striking 9-month increase in overall survival (OS).^12^

It is worth considering the large body of evidence for statins in cancer prevention or treatment, which received significant interest during major conferences held in 2015 and 2016 (ASCO, ASCO-GI, and the San Antonio Breast Cancer Symposium). Using large prospective cohort or registry studies, all but one study was positive. The magnitude of the effects found by these studies, along with study size, show why they attracted attention: a 22% reduction in cancer deaths among 146,326 women,^13^ no effect on breast cancer incidence among 79,518 women,^14^ 40% reduction in prostate deaths among 22,110 high-risk patients with prostate cancer,^15^ 14% reduction in all-cause mortality in 2,142 patients with pancreatic cancer,^16^ 18% reduction in recurrence and/or deaths in 8,010 patients with breast cancer,^17^ and 29% reduction in breast cancer mortality from a meta-analysis of 12 studies covering 87,951 patients with breast cancer.^18^ There have also been other large studies that have reported that statins, usually when still taken after diagnosis, can reduce recurrence or mortality in patients with esophageal,^19^ colorectal,^20^ and lung cancer,^21^ and all tumors combined,^22^ with further evidence from meta-analyses of all cancers,^23^ and prostate (postradiotherapy)^24,25^ and colorectal cancer.^26^ However, all studies were observational and none established a dose-response for statin efficacy.

We investigated statins for the treatment of SCLC because patients are treated with platinum and/or etoposide chemotherapy, used in the earlier experimental work, and the poor prognosis made it a good candidate for an inexpensive therapy even with a modest effect.

## PATIENTS AND METHODS

Etoposide and Cisplatin or Carboplatin as First-Line Chemotherapy With or without Pravastatin in Treating Patients with Small-Cell Lung Cancer (LUNGSTAR) was a pragmatic, randomized (1:1), phase III, double-blind, placebo-controlled trial to investigate whether adding pravastatin to standard chemotherapy improves OS in patients with SCLC. Investigators are listed in Appendix Table A1 (online only). The trial had national ethics approval and was conducted according to the Declaration of Helsinki. All patients gave written informed consent.

### Patients

Patients age ≥ 18 years were recruited from 91 United Kingdom National Cancer Research Network hospitals. Eligibility criteria included histologically or cytologically confirmed SCLC (limited or extensive disease), Eastern Cooperative Oncology Group performance status 0 to 3, life expectancy > 8 weeks, and adequate renal and bone marrow function. Patients were ineligible if they had mixed cell histology, prior chemoradiotherapy for the tumor, history of a malignant tumor, had used statins within the previous 12 months, or had been treated with fibrates within 4 weeks before random assignment. Patients were randomly assigned by research nurses after telephoning the University College London Cancer Trials Centre, using minimization stratified by disease status (limited *v* extensive) and Eastern Cooperative Oncology Group performance status (0 to 1 *v* 2 to 3).

Pravastatin or matching placebo (40 mg) were administered orally once per day from the start of chemotherapy for 2 years, unless disease progression or intolerable toxicity occurred. We used pravastatin because this seemed to be active in liver cancer^12^ and, unlike other statins, did not interact with cytochrome P450 family members, thereby reducing potential important drug interactions.^27,28^ All patients received standard treatment every 3 weeks for up to six cycles: etoposide (120 mg/m^2^ intravenously on day 1, then either the same on days 2 and 3 or 100 mg twice per day orally on days 2 and 3), with either cisplatin (60 mg/m^2^ intravenously on day 1) or carboplatin (on day 1: area under curve [AUC] 5 or 6 using EDTA, or the Cockcroft and Gault method, to assess glomerular filtration rate). Radiotherapy was administered per local practice. Patients with limited disease were recommended to have concurrent chemoradiotherapy, preferably with the second chemotherapy cycle, or sequential chemoradiotherapy to the chest (the protocol did not specify dose), with prophylactic cranial radiation offered to those who achieved a partial response or complete tumor response (CR). Patients with extensive disease and who achieved CR were offered thoracic radiotherapy—recommended 40 Gy in 15 fractions over 3 weeks to the mediastinum and 25 Gy in 10 fractions over 2 weeks to the brain. All researchers and patients were blinded to statin and placebo. Primary care physicians of randomly assigned patients were contacted and asked not to prescribe statins while their patient was enrolled in the trial—so we did not collect data on statin use outside of the study.

### Assessments

Clinical examinations, biochemical tests, and chest x-rays were performed at baseline, before each chemotherapy cycle, then every 2 months for the next year and every 3 months thereafter. Chest and abdomen computed tomography scans were performed at baseline, at the end of cycle 3, within 4 weeks of completing chemotherapy, and when clinically indicated thereafter. Brain scans were performed before random assignment, where indicated, to exclude patients with brain metastases who required immediate radiotherapy.

### Statistical Analysis

The primary end point was OS, measured from the date of random assignment until death from any cause. Surviving patients were censored on the date last known to be alive. Secondary end points were progression-free survival (PFS), tumor response assessed by the treating clinician (Response Evaluation Criteria in Solid Tumors [RECIST] v1.0) and toxicity (Common Terminology Criteria for Adverse Events v3.0). PFS was calculated from the date of random assignment to the date of first progression or death, whichever occurred first. OS and PFS were compared by using Cox proportional hazards regression models, adjusted for the randomization stratification factors, which were also preplanned subgroup analyses. Tablet adherence was assessed by a Wilcoxon test. The worst grade of adverse event for each patient and each toxicity type was used. All analyses were by intention-to-treat, except for adverse events, which were reported only for patients who took at least one dose of statin or placebo.

The trial was designed to detect an improvement in median OS with hazard ratio (HR) of 0.82 from an expected median in controls of 12 months, which corresponds to a difference in 2-year OS rates of 10% versus 15%. This required 842 patients (792 deaths) with 80% power and 5% two-sided significance.

## RESULTS

**Fig 1.**
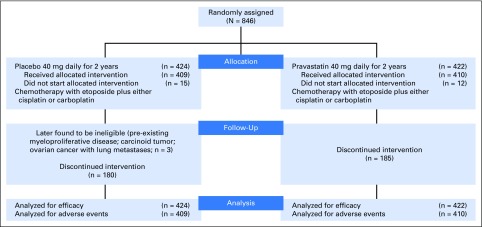
CONSORT diagram. Discontinued intervention includes patients who stopped statin and/or placebo early as a result of disease progression, toxicity, or patient and/or clinical decision.

Eight hundred forty-six patients were recruited between February 19, 2007 and January 3, 2012 (Fig 1). Of 1,537 patients who were screened, where screening logs were available, 338 (22%) patients were ineligible because they were recent or current statin users. Three randomly assigned patients who were later found to be ineligible were included in the analyses (intention-to-treat), because they had already started trial drug, which stopped within 6 months. Baseline characteristics were well balanced (Table 1). Median follow-up was 39.6 months.

**Table 1.**
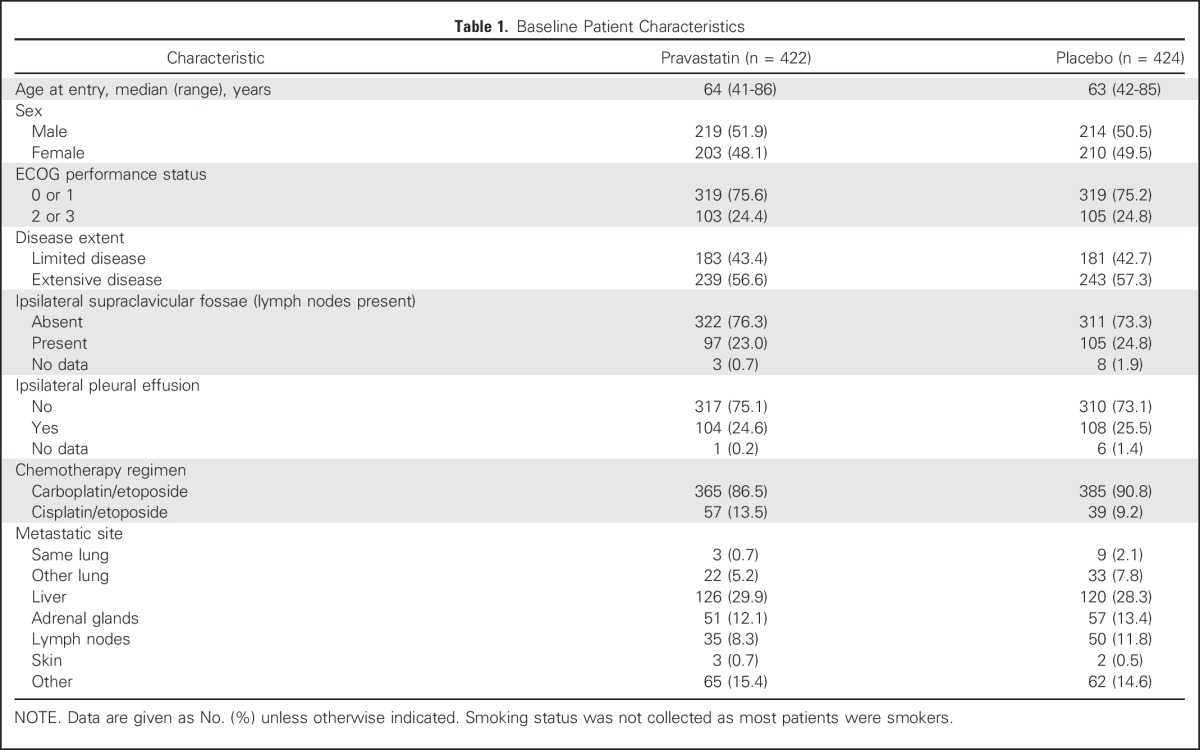
Baseline Patient Characteristics

### Adherence

The median length of time on study drug was 8.6 months (pravastatin) and 7.8 months (placebo). Among 725 patients who started treatment and with available data on number of tablets dispensed and returned, the median number of tablets reportedly taken was 210 (pravastatin) and 181 (placebo; *P* = .38). Similarly, there was little difference found in the number of tablets returned (median, 38 and 36, respectively).

The amount of chemotherapy administered was similar between the two groups (*P* = .19); in each treatment arm, 57% received six cycles (Appendix Table A2, online only). There was little difference in the reasons for stopping chemotherapy early (Appendix Table A3, online only). The mean number of chemotherapy cycles was similar between patients with extensive disease (4.8) and limited disease (4.9).

Appendix Table A4 (online only) summarizes the types of additional treatments administered to patients after they finished chemotherapy, which were well balanced between the statin and placebo arms, and within patients with extensive and limited disease.

### Efficacy

There were 758 deaths. Six hundred ninety-seven (92%) were a result of SCLC, including six deaths that were considered to be related to chemotherapy in the pravastatin group and none in the placebo group; eight deaths were attributed to a combination of cancer and treatment in the pravastatin arm and six in the placebo arm.

**Fig 2.**
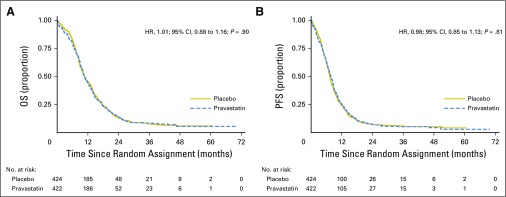
(A) Overall survival (OS) and (b) progression-free survival (PFS). The number of events in the pravastatin versus placebo groups were 381 versus 377 deaths, and 395 versus 392 PFS events, respectively. HR, hazard ratio.

OS was similar between treatment groups (Fig 2), with medians of 10.7 months and 10.6 months for pravastatin and placebo, respectively, (unadjusted HR, 1.01 [95% CI, 0.88 to 1.16; *P* = .90] and adjusted for the stratification factors [1.02; 95% CI, 0.89 to 1.18; *P* = .76]). The corresponding 2-year OS rates were 14.1% (95% CI, 10.9 to 17.7) and 13.2% (95% CI, 10.0 to 16.7), respectively.

There were 787 PFS events. Median PFS was 7.7 months (pravastatin) versus 7.3 months (placebo), with unadjusted HR of 0.98 (95% CI, 0.85 to 1.13; *P* = .81), and adjusted HR of 1.01 (95% CI, 0.88 to 1.17; *P* = .86). The corresponding 1-year PFS rates were 25.3% and 24.2%, respectively; the 2-year PFS rates were 7.5% (95% CI, 5.2 to 10.3) and 7.2% (95% CI, 4.9 to 10.0), respectively.

**Fig 3.**
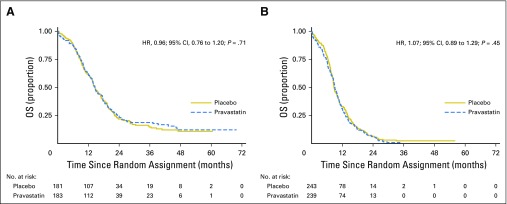
Overall survival (OS) in patients with (A) limited stage and (B) extensive stage disease. HR, hazard ratio.

**Fig A1.**
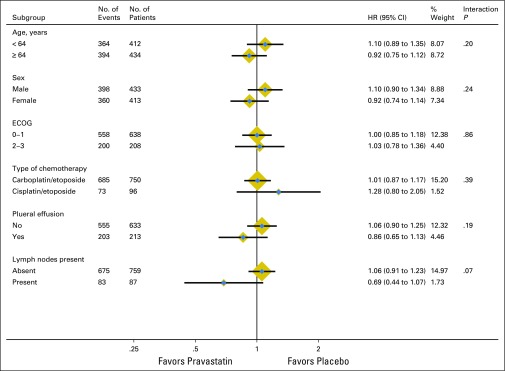
Forest plot showing subgroup analyses (overall survival). ECOG, Eastern Cooperative Oncology Group; HR, hazard ratio.

Pravastatin also had no effect as a function of disease extent (Fig 3). Median OS was 14.6 months (pravastatin) versus 14.6 months (placebo) for limited stage disease, and 9.1 months versus 8.8 months for extensive stage (interaction *P* = .53). Furthermore, no subgroup effects were observed for performance status, age, sex, type of platinum therapy administered, pleural effusion, or presence of affected lymph nodes (Appendix Fig A1, online only). Allowing for a slight imbalance between the two arms for type of platinum treatment (Table 1) made little difference to the results (HR, 1.04; 95% CI, 0.90 to 1.19; *P* = .63).

Tumor response was similar between trial groups with 29 (69.0%) of 422 patients on pravastatin and 293 (69.1%) of 424 patients on placebo achieving a partial or complete (best) overall response; there was little difference in response rates for limited and extensive stage disease (Appendix Table A5, online only).

The number of patients who received thoracic radiotherapy was similar between trial groups with 202 (47.9%) patients in the pravastatin arm and 210 (49.5%) in the placebo arm, with a corresponding median total dose of 39 Gy (range, 3 to 66) and 40 Gy (range, 2 to 66), respectively. Furthermore, 203 (48.1%) patients in the pravastatin arm and 207 (48.8%) patients in the placebo arm received prophylactic cranial brain irradiation, with corresponding median total dose of 25 Gy (range, 2 to 40) and 25 Gy (range, 2 to 56), respectively.

### Adverse Events

Table 2 shows that the distribution of grade 3 to 5 adverse events was similar between the pravastatin and placebo arms with 333 (81.2%) of 410 patients versus 333 (81.4%) of 409 patients, respectively (*P* = .94). The most common grade 3 to 5 adverse event was neutropenia, which affected 184 (44.9%) of patients in the pravastatin arm and 176 (43.0%) of patients in the placebo arm. Myalgia or myositis of any grade—recognized toxicities of statins—occurred in 74 patients in the pravastatin arm and 77 patients in the placebo arm; the majority of these were grade 1 or 2, with three patients who received pravastatin experiencing grade 3 and 4 (three grade 3, one grade 4) compared with three patients who received placebo (all grade 3). GI bleeding (grade 1) was experienced by three patients in the statin group, in addition to two (grade 2) and one (grade 3) events. GI bleeding (grade 1) was experienced by two patients in the placebo group in addition to two (grade 2) events.

**Table 2.**
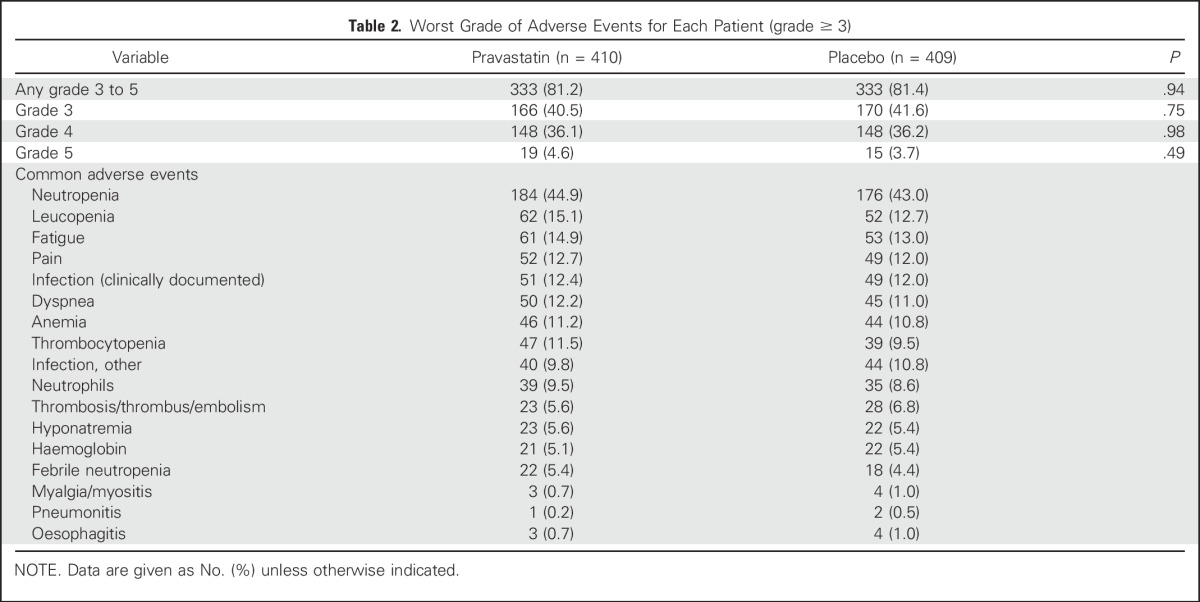
Worst Grade of Adverse Events for Each Patient (grade ≥ 3)

## DISCUSSION

To our knowledge, LUNGSTAR, by far, is the largest randomized trial of statin therapy in patients with cancer reported to date. Use of placebo avoids an important bias present in observational studies. Although pravastatin was safe, with high adherence—patients tended to continue until disease progression—it did not improve outcomes in patients with SCLC, nor in those with limited stage or extensive stage disease.

When LUNGSTAR was developed in 2005, preclinical evidence was sufficient, though not considered overwhelming by some; however, rather than perform further experimental and/or preclinical studies, followed by phase II, then phase III trials, the investigators and funder (Cancer Research UK) decided to launch a definitive large study sooner rather than later, given the growing evidence in this field at the time. The independent data monitoring committee reviewed efficacy during the trial. In April 2010, when 25% of the target number of deaths was observed, conditional power (CP) for OS was 66%—CP is the chance of obtaining the target HR of 0.82 if the trial continued to the end, given the data thus far. In February 2011, with 655 patients recruited and 41% of events, CP was 11% for OS, but 30% for PFS, which was not considered low enough to stop the study early, even though it was a secondary end point. Additional important reasons for finishing accrual were to have a large study size with convincing results, whether positive or negative, particularly given the accumulating positive observational studies, and to ensure sufficient patient numbers for limited disease and extensive disease in case pravastatin was effective for one and not the other.

Various possibilities might explain our findings, including dose, type of statin, or that our mechanistic understanding was too simplistic. When our study was established, the maximum pravastatin dose was 40 mg, which seemed to be effective in a randomized cancer trial^12^; however, this dose might be too low and the current 80 mg maximal dose could have achieved efficacy. Alternatively, hydrophilic statins, such as pravastatin, may not be as effective as lipophilic agents, such as simvastatin; some studies have suggested clearer benefits for simvastatin in lung cancer,^21^ with a lack of benefit for pravastatin in cancer prevention or cancer death observed in a trial of patients with coronary heart disease.^29^ However, there is insufficient evidence to reliably conclude whether any one type of statin is better than another, and biologic plausibility for a difference is lacking.^22^ Of interest, more recent evidence in glioma cells has suggested that statins may fail to work in certain cancer cells because of a phosphatidylinositol 3-kinase–mediated pathway connected to LDL receptors.^30,31^ It is unclear whether this might impact the responsiveness to statins of other cancers. Another study limitation is that blood lipid levels were not measured as part of routine biochemistry for managing patients with SCLC, which would have unblinded the trial, and we did not secure funds to measure cholesterol and other relevant markers from stored samples; therefore, we are unable to correlate these or other factors, such as HMG-CoA reductase levels, in tumor biopsies with outcomes at present.

We compared patient outcomes in LUNGSTAR with others. Our observed median OS (10.7 months) is similar to that observed in another United Kingdom trial of SCLC (comparing thalidomide with placebo, but little difference was found), which had a similar mix (approximately one half) of limited stage and extensive stage disease (median 10.5 months).^32^ However, survival for limited stage patients (median 14.6 months LUNGSTAR, 12.1 months in the thalidomide trial), is less than observed in a recent radiotherapy trial (median OS, 25 to 30 months),^33^ probably because it enrolled patients from several countries, where more radiotherapy has been given than in the United Kingdom in previous years.

We conducted a systematic literature review to identify all randomized trials that were specifically designed to evaluate lipid-lowering therapies among patients with cancer using MEDLINE (1966 to March 2016), and the keywords ‘lipid’, ‘cholesterol’, ‘statin’ (and specific names), and ‘tumor/tumor’, ‘cancer’, ‘carcinoma’, ‘adenocarcinoma’ and ‘random.’ There were only 10 trials^12,34-42^ (9 of statins), which are summarized in Table 3. LUNGSTAR is three times larger than any other randomized trial, the next largest having enrolled 283 patients. The 9-month OS improvement for pravastatin in the earlier trial by Kawata et al^12^ was probably the result of a lack of blinding and small study size (n = 83). Of importance, there are now five published double-blind studies of statins in patients with cancer, including ours (SCLC, gastric, pancreas, colorectal, and precancerous melanoma lesions), of which one used pravastatin, one lovastatin, and three simvastatin, and none showing statins of various types to be effective.^36-39^

**Table 3.**
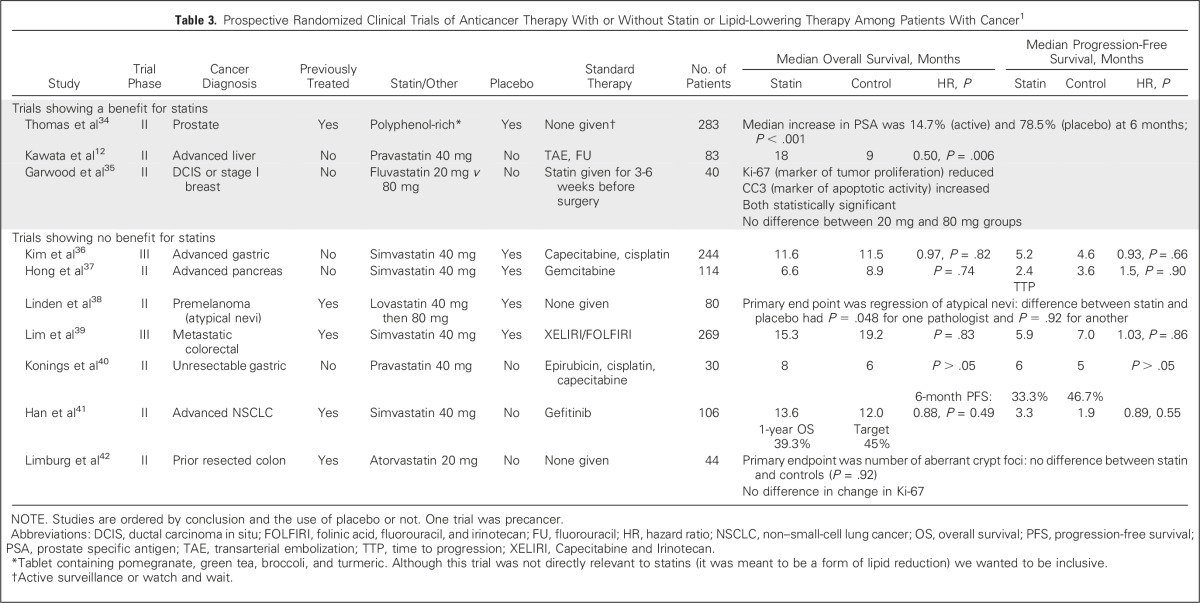
Prospective Randomized Clinical Trials of Anticancer Therapy With or Without Statin or Lipid-Lowering Therapy Among Patients With Cancer^1^

We also found a single-arm, phase II trial and a nonrandomized, unblinded trial in patients with lung cancer. One was for previously untreated extensive-disease– SCLC (n = 61) in which simvastatin plus irinotecan and etoposide produced a 1-year OS rate of 39.3% (target 45%); median OS and PFS were 11.0 months and 6.1 months, respectively, which was similar to current treatments.^43^ The other trial included patients with stage III and IV cancer with non-SCLC, whose tumors were KRAS-mutant and epidermal growth factor receptor wild-type, reported better outcomes for 12 patients treated with a tyrosine kinase inhibitor plus either simvastatin or atorvastatin compared with 55 who received tyrosine kinase inhibitor alone. In that study the PFS was 2.0 months versus 1.0 month (*P* = .025), and OS 14.0 months versus 5.4 months (*P* = .13); however, it was not reported why those patients received a statin, hence there could be important confounders not allowed for.^44^

Most prior studies that have reported benefits for statins were observational, with inherent design problems, including confounding and bias.^45^ Time-related biases are particularly concerning.^46^ Immortal time bias occurs when there is a length of time between the start of follow-up and a subsequent start of statin therapy, which is counted in the total follow-up time for a participant—but during which the participant is actually unexposed—and, thus, it seems that this participant has survived longer than a control (nonstatin user). Jeon et al^47^ show how this bias can spuriously create an association by using studies of patients with liver cancer in which the OS HR for statin use versus nonuse was 0.84 (*P* = .047) before and 0.98 (*P* = .82) after allowance for this bias. Randomized controlled trials, such as LUNGSTAR, avoid these issues.

Preclinical studies continue to report positive effects for statins in lung cancer cell lines with regard to reduced proliferation,^48,49^ reduced migration,^50^ increased apoptosis,^49^ and reduced tumor growth.^50^ There are also several ongoing trials of statins in various cancers (eg, ClinicalTrials.gov: NCT02360618, NCT01980823, NCT01038154, NCT02161822, NCT02483871, NCT02569645, and NCT02029573). Given the findings from our trial and the other published, double-blind, randomized controlled trials, independent data monitoring committees of studies that are still recruiting or in follow-up should examine interim analyses of clinical end points and stop early if there is sufficient evidence for futility, thus saving resources. Trials of statins in patients with cancer, which require an unexposed control group, will become more difficult to conduct because the usual age group of patients with cancer (middle and old age) already take them, as seen in LUNGSTAR, in which 22% of screened patients were ineligible because they were recent or current statin users.

In summary, we found no value for pravastatin when combined with standard platinum chemotherapy in patients with SCLC. Ongoing and future trials of statins used for either cancer prevention or treatment should monitor clinical efficacy, and for planned studies of patients with cancer, investigators should ensure preclinical evidence is sufficient enough to warrant large-scale randomized studies.

## Figures and Tables

**Table A1.**
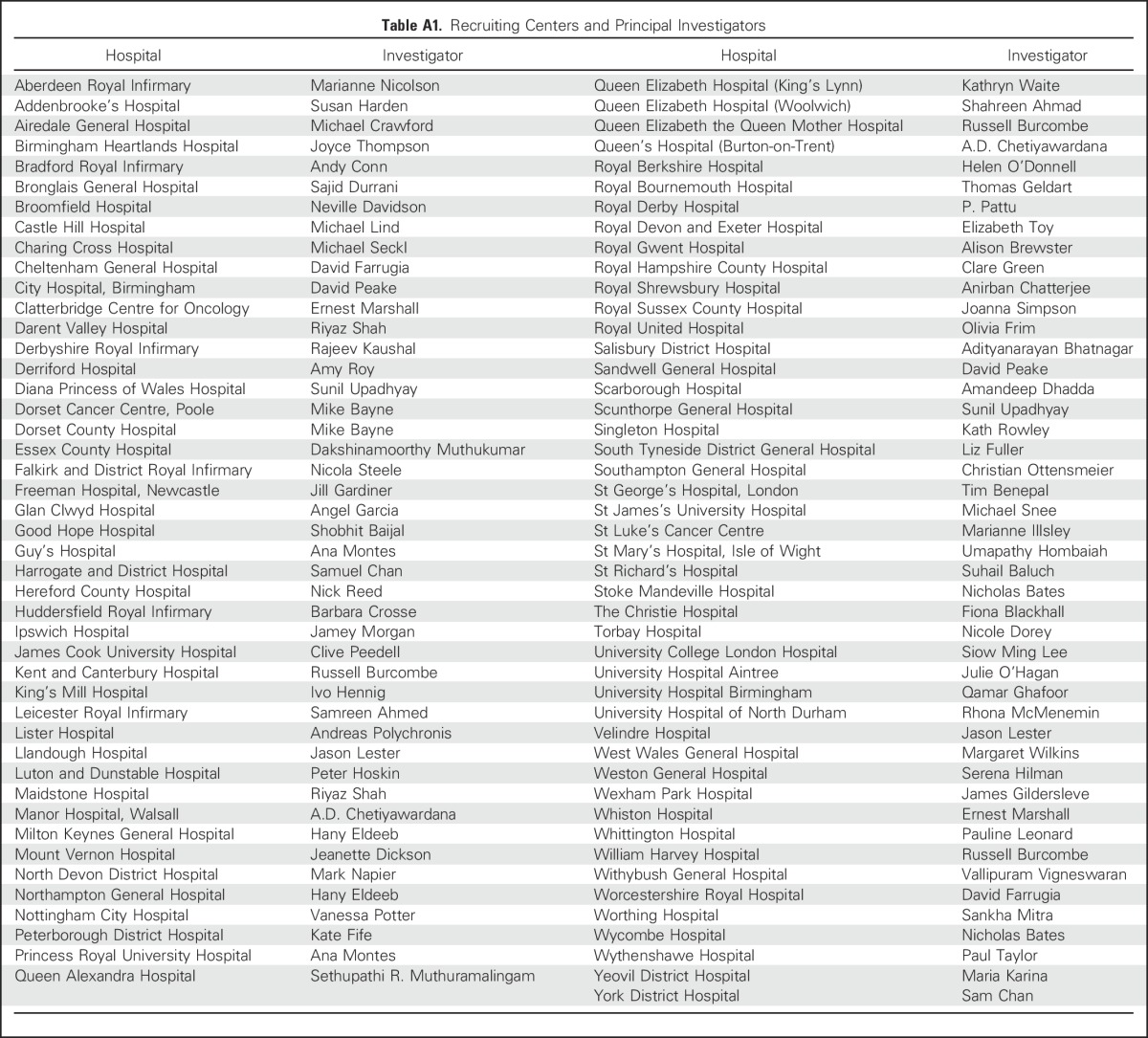
Recruiting Centers and Principal Investigators

**Table A2.**
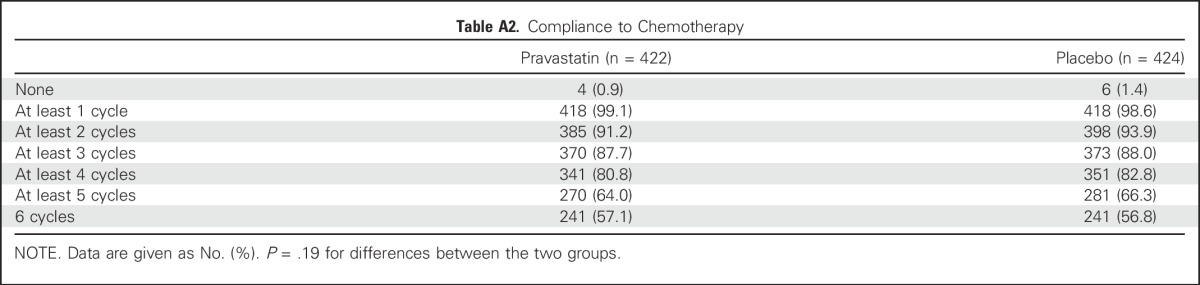
Compliance to Chemotherapy

**Table A3.**
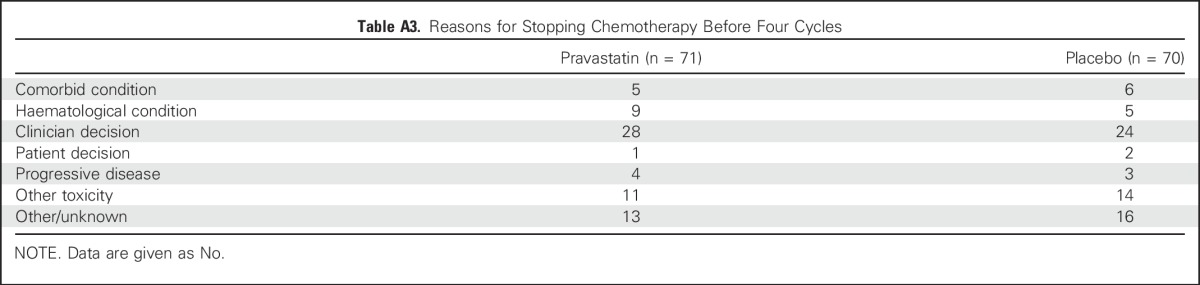
Reasons for Stopping Chemotherapy Before Four Cycles

**Table A4.**
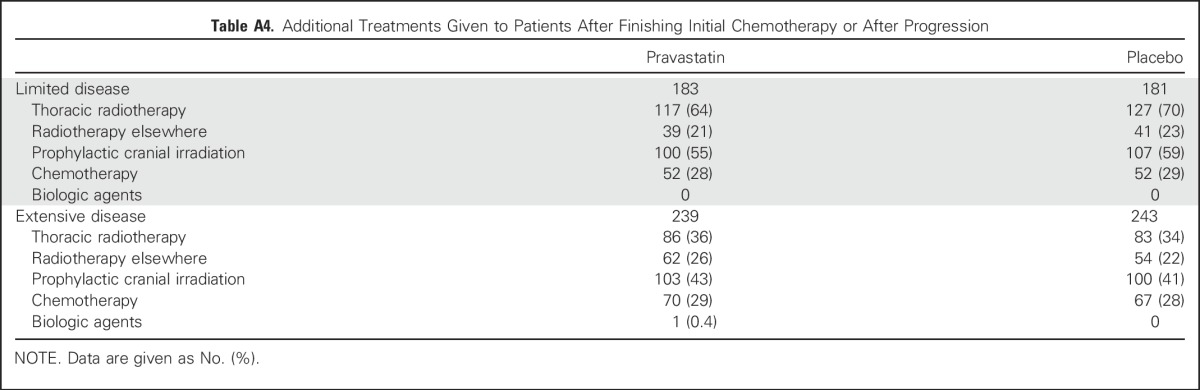
Additional Treatments Given to Patients After Finishing Initial Chemotherapy or After Progression

**Table A5.**
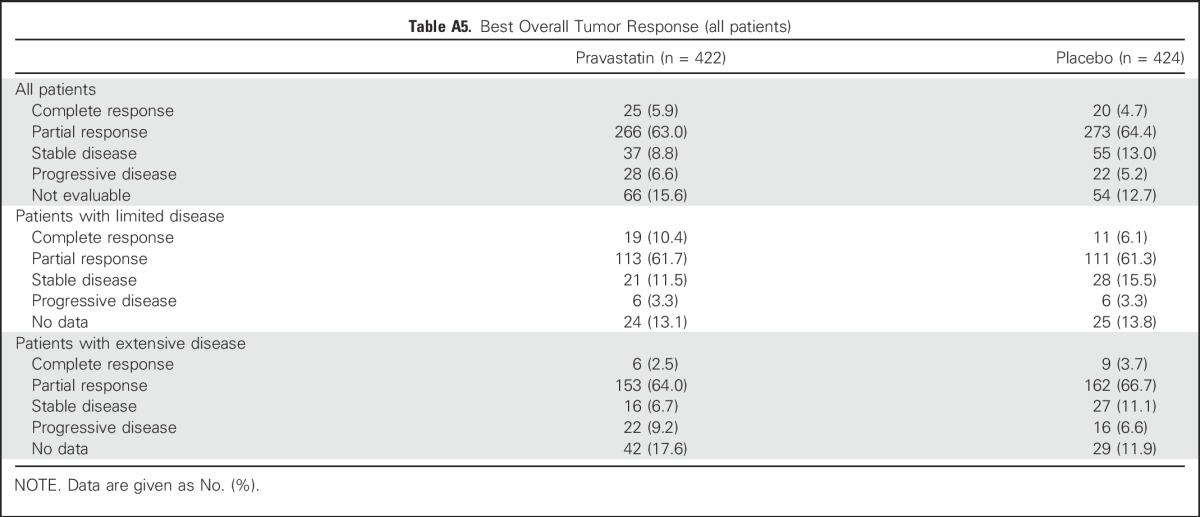
Best Overall Tumor Response (all patients)
